# First whole genome sequencing and analysis of human parechovirus type 3 causing a healthcare-associated outbreak among neonates in Hungary

**DOI:** 10.1007/s10096-024-04950-4

**Published:** 2024-09-27

**Authors:** Nóra Deézsi-Magyar, Nikolett Novák, Adrienne Lukács, Katalin Réka Tarcsai, Ágnes Hajdu, László Takács, Ferenc Balázs Farkas, Zita Rigó, Erzsébet Barcsay, Zoltán Kis, Katalin Szomor

**Affiliations:** 1 Department of Microbiological Reference Laboratories, National Center for Public Health and Pharmacy, Albert Flórián Rd. 2-6. 1097, Budapest, Hungary; 2 Department of Communicable Disease Epidemiology and Infection Control, National Center for Public Health and Pharmacy, Budapest, Hungary; 3 Bethesda Children Hospital, Budapest, Hungary; 4 Pediatric Center, Semmelweis University, Budapest, Hungary; 5 School of PhD Studies, Semmelweis University, Budapest, Hungary; 6 Institute of Medical Microbiology, Faculty of Medicine, Semmelweis University, Budapest, Hungary

**Keywords:** Human parechovirus 3, Infection of neonates, Whole genome sequencing, Genotyping, Phylogenetic analysis

## Abstract

**Purpose:**

In November 2023, the National Reference Laboratory for Enteroviruses (Budapest, Hungary) received stool, pharyngeal swab and cerebrospinal fluid samples from five newborns suspected of having human parechovirus (PEV-A) infection. The neonates were born in the same hospital and presented with fever and sepsis-like symptoms at 8–9 days of age, and three of them showed symptoms consistent with central nervous system involvement. PEV-A positivity was confirmed by quantitative reverse transcription polymerase chain reaction.

**Methods:**

To determine the PEV-A genotype responsible for the infections, fecal samples of four neonates were subjected to metagenomic sequencing. For further analyses, amplicon-based whole genome sequencing was performed directly from the clinical samples.

**Results:**

On the basis of whole genome analysis, sequences were allocated to PEV-A genotype 3 (PEV-A3) and consensus sequences were identical. Two ambiguities were identified in the viral protein 1 (VP1) region of all sequences at a frequency of 17.7–53.7%, indicating the simultaneous presence of at least two quasispecies in the clinical samples. The phylogenetic analysis and similarity plotting showed that all sequences clustered without any topological inconsistencies between the P1 capsid and P2, P3 non-capsid regions, suggesting that recombination events during evolution were unlikely.

**Conclusion:**

Our findings suggest that the apparent cluster of cases were microbiologically related, and the results may also inform future investigations on the evolution and pathogenicity of PEV-A3 infections.

**Supplementary Information:**

The online version contains supplementary material available at 10.1007/s10096-024-04950-4.

## Introduction

Human parechoviruses (PEV-A) are non-enveloped viruses with icosahedral symmetry belonging to the family *Picornaviridae*, genus *Parechovirus*, species *Parechovirus A* [1, 2]. The single-stranded positive-sense RNA genome is approximately 7300 nt bases in length and encodes a single polyprotein in a single open reading frame (ORF) with untranslated regions (UTRs) at the 5’ and 3’ termini of the genome [2]. The polyprotein is post-translationally cleaved by the 3 C viral protease to generate the mature structural viral proteins (VP0, VP3 and VP1) and seven non-structural proteins (2 A, 2B, 2 C and 3 A, 3B, 3 C and 3D) [2, 3]. In children under 2 years of age, PEV-A is a common pathogen, causing mostly asymptomatic infection or mild respiratory and/or gastrointestinal symptoms [4, 5]. However, primary PEV-A infection has been associated with encephalitis, meningitis, acute flaccid paralysis, necrotizing enterocolitis, hepatitis and sepsis syndrome in neonates and infants under the age of 3 months [5, 6]. In this age group, PEV-A is the second most common causative agent of central nervous system (CNS) viral infections after enteroviruses [5]. The main mode of transmission is the fecal-oral route [7, 8].

Routine microbiological diagnostic procedures include the detection of nucleic acid directly from the respiratory and/or fecal samples of the individual by quantitative reverse transcription polymerase chain reaction (RT-qPCR) targeting the 5’ UTR region of the genome [9, 10]. Infected individuals can shed the virus in their feces for up to several months [7, 8]. In cases of CNS involvement, cerebrospinal fluid (CSF) samples can also be used for diagnosis [9, 10]. Virus isolation, a commonly used method for enterovirus diagnosis, is less sensitive to PEV-As, as they show moderate and genotype-dependent adaptation to commonly used cell lines, such as RD, Vero and A549 [5, 10].

To date, 19 PEV-A genotypes (PEV-A1-19) have been identified based on the VP1 capsid region [1, 11]. Due to the lack of proof-reading mechanism of the RNA-dependent RNA polymerase encoded by the 3D gene, PEV-As show high viral diversity, with a 30% and 15% divergence at nucleotide and amino acid levels, respectively, with high mutation rates of approximately 2.5 × 10^− 3^ substitution/site/year [8, 11, 12]. Analyses of complete PEV-A genomes highlighted increased recombination among inter- and intra-genotypes, with recognized breakpoints between the 5’ UTR/P1 and P2/P3 junctions, increasing overall virus diversity; however, breakpoints within the VP1 region are rare [2, 4, 11, 12].

PEV-A infections commonly occur in the general population; approximately 95% of the adult population is seropositive for PEV-A1-specific antibodies. Infections are often acquired in early childhood by the age of 18 months and with 20% of children being infected after the first year of life [13, 14]. The most common genotypes accounting for the majority of infections are PEV-A3, 1, 6, 4, 5 and 14, whereas PEV-A3 and 5 are the most frequently involved in severe neurological illness caused by PEV-A in neonates [8, 11, 15, 16].

The clinical and public health importance of PEV-A was recognized in the last decade and since then, routine diagnosis and differential diagnosis of enteroviruses have been continuously improved [16]. Consequently, with the increasing availability of laboratory tests, the number of PEV-A diagnoses has increased compared with previous years [9, 16].

There are multiple estimates on the worldwide spread of PEV-A. Studies in Europe and the US suggest PEV-A prevalence of 1–7% with the most prevalent genotypes being PEV-A3 followed by 1, 6, 4 and 5, and in some parts of Asia, PEV-A prevalence is estimated to be as high as 25% [4, 9, 14, 17, 18]. In May 2022, a multi-state PEV-A3 epidemic among neonates and young infants was reported in the US [9]. In Hungary, PEV-A1 and PEV-A4 were previously detected in archived isolates and published in 2010. Since then, PEV-A3 has also been identified based on partial sequencing of the VP1/VP3 genes directly from clinical samples of symptomatic children [19, 20]. However, no further data on virus circulation in the country are available.

## Materials and methods

### PEV-A cases and clinical samples

In November 2023, stool, pharyngeal swabs (washed into virus transport medium) and CSF samples from four neonates (Neonates 1–4) with suspicion of PEV-A infection were sent to the National Reference Laboratory (NRL) for Enteroviruses, Budapest. The neonates were born in Hospital X on two adjacent days, and discharged with their respective mothers without any medical issues. At the age of 8–9 days, the neonates were taken to the emergency department of the hospital with high fever and sepsis-like symptoms. One neonate (Neonate 2) was admitted to the Neonatal and Paediatric Department of Hospital X. Three neonates (Neonates 1, 3 and 4) also presented CNS symptoms, and two of them (Neonates 1 and 4) were transferred to a Children’s Hospital in Budapest for intensive care, one of them requiring invasive respiratory support and vasopressor therapy. Neonate 3 was transferred to another Pediatric Center in Budapest, for suspected necrotizing enterocolitis (which was not confirmed), and later developed mild, transient CNS symptoms. PEV-A infection was confirmed in all four cases from all sample types using RT-qPCR. Contact persons were also screened primarily from pharyngeal swab samples (including mothers of confirmed neonatal PEV-A cases, contact neonates and health-care workers). Seven days after these four cases were identified, one 18-day old neonate (Neonate 5) – who shared a rooming-in ward with two confirmed neonatal PEV-A cases (Neonates 1 and 4) in Hospital X when they were born – also tested positive for PEV-A. This fifth neonatal PEV-A case received outpatient treatment for urinary tract infection, and – due to the increased awareness of neonatal PEV-A infection by that time – a pharyngeal swab sample was taken during a routine check-up at Hospital X and sent to the NRL for Enteroviruses.

### Laboratory diagnosis of PEV-A

**Fig. 1:**
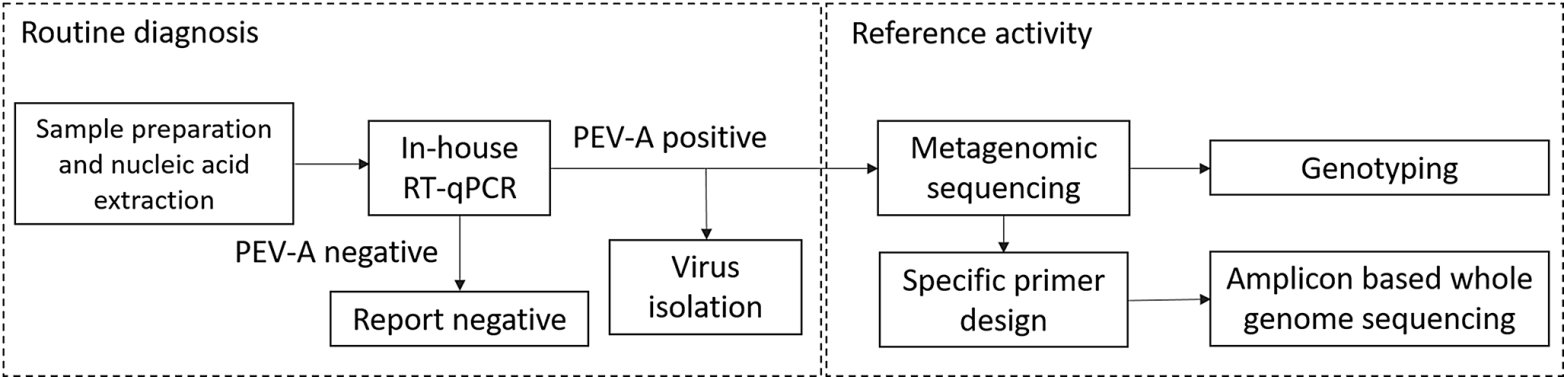
PEV-A routine diagnostic procedure followed by genotyping and genome analysis using metagenomic sequencing and amplicon-based whole genome sequencing (National Reference Laboratory for Enteroviruses, National Center for Public Health and Pharmacy, Budapest, Hungary)

The laboratory procedure of PEV-A routine diagnosis followed by genotyping and amplicon-based whole genome sequencing is shown in Fig. 1. Fecal samples, prior to nucleic acid extraction were washed into Dulbecco modified Eagle medium (DMEM; VWR International bv, Leuven, Belgium) supplemented with 1% antibiotics. Viral RNA from clinical samples was extracted using the QIAamp Viral RNA Mini Kit (Qiagen GmbH, Hilden, Germany) according to the manufacturer’s instructions. Subsequent RT-qPCR was performed at the LightCycler 480 Instrument II platform (Hoffmann-La Roche, Basel, Switzerland) using the LightCycler Multiplex RNA Virus Master kit (Hoffmann-La Roche, Basel, Switzerland). The applied primers and probes specific for the 5’ UTR genome region were previously described by Lu et al. [21].

### Metagenomic and amplicon-based sequencing

Viral nucleic acid extracted directly from the fecal samples of the initial four neonatal PEV-A cases were subjected to metagenomic sequencing starting with DNase I digestion (Turbo DNAse; Invitrogen, Thermo Fisher Scientific, Waltham, Massachusetts, USA) and AMPure XP bead (Beckman Coulter, Brea, California, USA) purification. Clear nucleic acid was eluted in 10 µL nuclease-free water. To reduce the abundance of host-derived ribosomal RNA, the RiboCop rRNA Depletion Kit HMR V2 (Lexogen GmbH, Vienna, Austria) was used according to the manufacturer’s instructions. Randomly amplified DNA products were prepared using the sequence-independent single primer amplification (SISPA) protocol with the RA01/RA01-N8 primers as previously described [22, 23]. After enrichment, the Illumina Nextera XT V2 (Illumina, Waltham, Massachusetts, USA) library preparation was performed. Sequencing was conducted on the Illumina MiSeq (Illumina, Waltham, Massachusetts, USA) instrument using 2 × 150 bp paired-end chemistry (Reagent Kit v2 Micro; Illumina, Waltham, Massachusetts, USA).

To amplify whole genome sequences, a custom-made panel of specific primers was designed using the PrimalScheme 1.4.1 web application [24] by setting the amplicon size to 1000 bp (Supplement Table 1). As input reference the PEV-A3 isolate under the Genbank accession number KY556671.1 was used.

Selected PEV-A positive samples with Ct values < 33.1 were subjected to amplicon based whole genome sequencing. After DNAse I digestion and AMpure XP bead purification, pure nucleic acid was eluted in 10 µL nuclease-free water. Reverse-transcription was performed using the SuperScript™ VILO™ cDNA Synthesis Kit (Invitrogen, Thermo Fisher Scientific, Massachusetts, USA). To generate the amplicons, PCR products were amplified in two pools using the Phusion^®^ High-Fidelity DNA Polymerase enzyme (New England Biolabs, Ipswich, England) and Phusion^®^ HF Buffer supplemented with the custom-designed primers (Supplementary Table S1). After initial denaturation at 98 °C for 30 min, 40 cycles of PCR amplification were performed including denaturation at 98 °C for 10 s, annealing at 52 °C for 20 s and extension at 72 °C for 40 s. After enrichment, the library preparation and sequencing protocols were performed according to the same method as described above.

### Genome assembly

To assemble the genome sequences, *de novo* and reference mapping were performed using Geneious Prime 2021.2.2 (Biomatters Inc, Auckland, New Zealand) and Genome Detective Virus Tool [25]. Raw reads were trimmed by the BBDuk plug-in with minimum quality of 20 and minimum sequence length of 30 bp, and duplicate reads were removed. Reference mapping was performed against the KY556671.1 complete genome sequence. Consensus sequences were called with a threshold of bases corresponding at least 40% of the read sequences. Low frequency nucleotide variations within the coding region were called with a minimum of 15% frequency and coverage of 1000x using the Geneious Prime 2021.2.2 software.

### Phylogenetic analysis

Nucleotide alignment was performed using the Clustal Omega 1.2.2 multiple alignment tool installed in Geneious Prime 2021.2.2 software [26]. Complete PEV-A genomes (*n* = 33 sequences) from the Genbank were downloaded to perform genotype classification based on the full-length coding region.The Maximum Likelihood method of MEGA X v10.0.5 software was used to determine the best-fit model of nt substitution [27]. For phylogenetic tree construction, the general time reversible model with gamma distribution and invariant sites (GTR + G + I) was performed [27, 28]. Phylogenetic analysis based on VP1 and VP3 regions was also performed using the Tamura-3-parameter model with gamma distribution (T92 + G) including closely related PEV-A3 sequences and previously described Hungarian VP1 (*n* = 1) and VP3 (*n* = 8) sequences [19]. Phylogenetic analysis of the P1, P2 and P3 coding regions was also conducted along with high percentage identity sequences obtained with the BLAST algorithm including the best 100 hits with query cover of 100%. Bootstrap support values were calculated using 1000 replicates. To estimate the average evolutionary distance of the P1, P2 and P3 regions, we used the Maximum Composite Likelihood method with 1000 bootstrap replications within the MEGA X v10.0.5 software.

### Recombination analysis

To confirm potential recombination events, similariry plotting was performed against recombinant and non-recombinant PEV-A sequences closely related to the newly obtained Hungarian strains, using Simplot + + v1.3, with a 200 bp window moving in 20 nt steps using the Kimura distance model.

## Results

### Microbiological diagnosis of PEV-A infection

During the outbreak investigation, a total of 56 samples (17 stool, 35 pharyngeal swabs and 4 CSF samples) from 32 individuals were tested for PEV-A. Key epidemiological and microbiological results of the five confirmed neonatal PEV-A cases and two asymptomatic PEV-A-positive mothers are shown in Tables 1 and 2, respectively. Ct values of the positive samples ranged between 29.64 and 34.02, with an average of 0.84 Ct lower in stool samples, indicating higher virus concentration compared to pharyngeal swabs. Virus isolation from positive samples was also attempted in Vero, Vero E6, RD and A549 cells, without success so far. During the course of the outbreak, no PEV-A positive cases other than the five neonates and the two mothers were detected among screened individuals. To the best of our knowledge, the affected neonates recovered without further complications, and their current psychomotor development is appropriate for their age.

**Table 1:** PEV-A-positive neonates identified: age, symptoms and obtained sample types with ct values and PEV-A genotype. *This sample was not tested at the National Reference Laboratory for Enteroviruses

Neonate	Age at onset of symptoms	Symptoms	Type of sample	Ct value	Genotype	Genbank accession number	Co-infections
Neonate 1	8 days	Fever, sepsis-like symptoms, CNS symptoms	Stool	31.11	PEV-A3	PP176215	*Klebsiella oxytoca*
			Pharyngeal swab	33.12	Not performed		
			Nasal swab	31.55	PEV-A3	PP176214	
			CSF 1. (lumbar puncture)	34.80	Not performed		
			CSF 2. (lumbar puncture)	34.82	Not performed		
Neonate 2	8 days	Fever, sepsis-like symptoms	Stool	29.64	PEV-A3	PP176212	*Klebsiella oxytoca*, *Enterococcus faecalis*
			Pharyngeal swab	32.71	PEV-A3	PP176213	
Neonate 3	9 days	Fever, sepsis-like symptoms	Stool	30.65	PEV-A3	PP176209	*Escherichia coli*,* Enterococcus faecalis*
			Pharyngeal swab	30.90	PEV-A3	PP176210	
			CSF (lumbar puncture)*	n/a	Not performed		
Neonate 4	8 days	Fever, sepsis-like symptoms, CNS symptoms	Stool	33.07	PEV-A3	PP176211	
			CSF (lumbar puncture)	32.10	PEV-A3	PP176217	
Neonate 5	18 days	Respiratory symptoms, fever, lethargy	Pharyngeal swab	32.11	PEV-A3	PP176216	Urinary tract infection of unknown origin

Metagenomic sequencing directly from the clinical specimens revealed partial PEV-A genome regions (17.8–49.8% genome coverage). BLAST analysis of the contigs and the Enterovirus genotyping tool [29] showed that all sequences were allocated to PEV-A genotype 3.

**Table 2:** Mothers of PEV-A-positive neonates: age, symptoms and sample types with ct values

Mothers	Age	Symptoms	Number of days between the neonate’s onset of symptoms and the sampling of the mother	Type of sample	Ct value
Mother of Neonate 1	34	Asymptomatic	5	Stool	32.30
				Pharyngeal swab	*negative*
Mother of Neonate 2	34	Asymptomatic	4	Pharyngeal swab	*negative*
Mother of Neonate 3	39	Asymptomatic	4	Pharyngeal swab	32.23
Mother of Neonate 4	29	Asymptomatic	4	Pharyngeal swab	*negative*

### Full-length genome analysis

For a more comprehensive genome analysis, we conducted the amplicon-based whole genome sequencing on samples (*n* = 9) from all five symptomatic neonates. Selected samples and genome assembly metrics are shown in Supplementary Table S2.

**Fig. 2:**
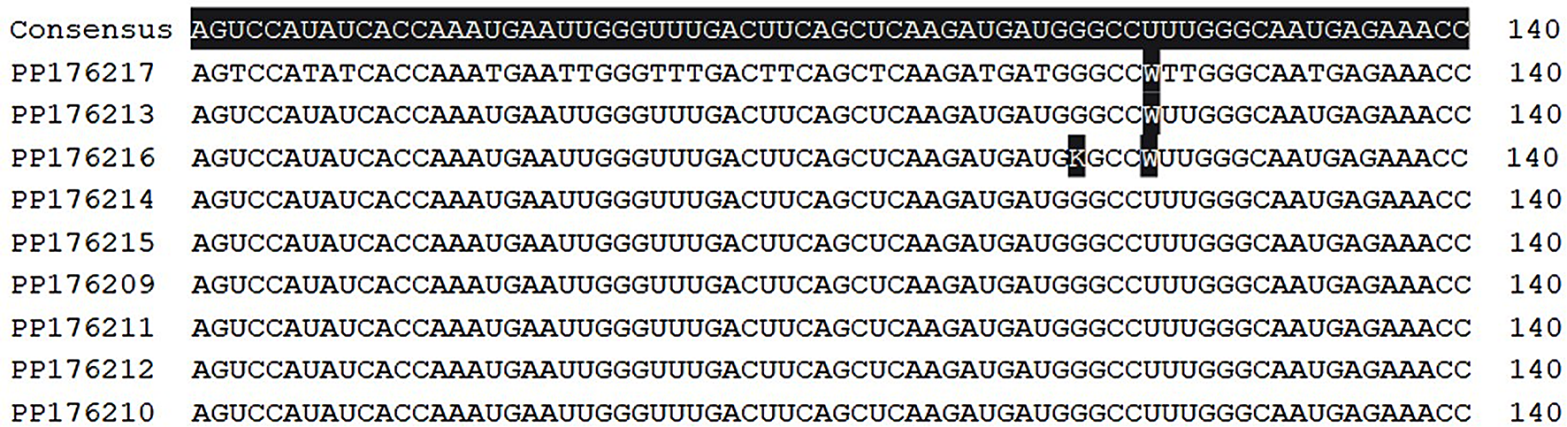
VP1 region, positions 71–140. Ambiguities in sequence PP176216 in positions 119 (G > U, 48.0%) and 123 (U > A, 53.7%) indicating the simultaneous presence of at least two quasispecies. Ambiguity (W) in position 123 is also present at a frequency over 40% in sequences PP176217 and PP176213, and at ≤ 40% frequency in the other sequences (software: Geneious Prime 2021.2.2.)

Consensus sequences were called with a threshold of bases matching at least 40% of the read sequences. At the consensus level, the overall genome composition was identical in all sequences; 31.6% A, 19.0% C, 20.8% G and 28.6% U, with GC content of 39.8%. Consensus sequences contained a 5’ UTR of 660 nt, a single ORF of 6534 nt encoding the VP0 (289 aa), VP3 (256 aa) and the VP1 (226 aa) capsid proteins, the 2 A (149 aa), 2B (122 aa), 2 C (329 aa), 3 A (117 aa), 3B (20 aa), 3 C (200 aa) and 3D (469 aa) non-structural proteins, and a 3’ UTR of 99 nt. In sequence PP176216 (Neonate 5, pharyngeal sample), there were ambiguities at the consensus level in nt positions 119 (G > U, 48.0% frequency) resulting in Gly to Val amino acid changes in aa position 40, and 123 (U > A, 53.7% frequency, synonymous mutation) of the VP1 region indicating the simultaneous presence of at least two quasispecies in the sample (Fig. 2). In the other genome sequences, G > U change in position 119 was also present at a frequency of 17.7–38.4%, and the U > A transversion in position 123 was persisting at 22.0–41.1% frequency. When the different sample types of the patients were compared, no deviations in the variant frequency were observed, suggesting that no intra-host adaptation during infection occur (data not shown).

### Phylogenetic analysis

**Fig. 3:**
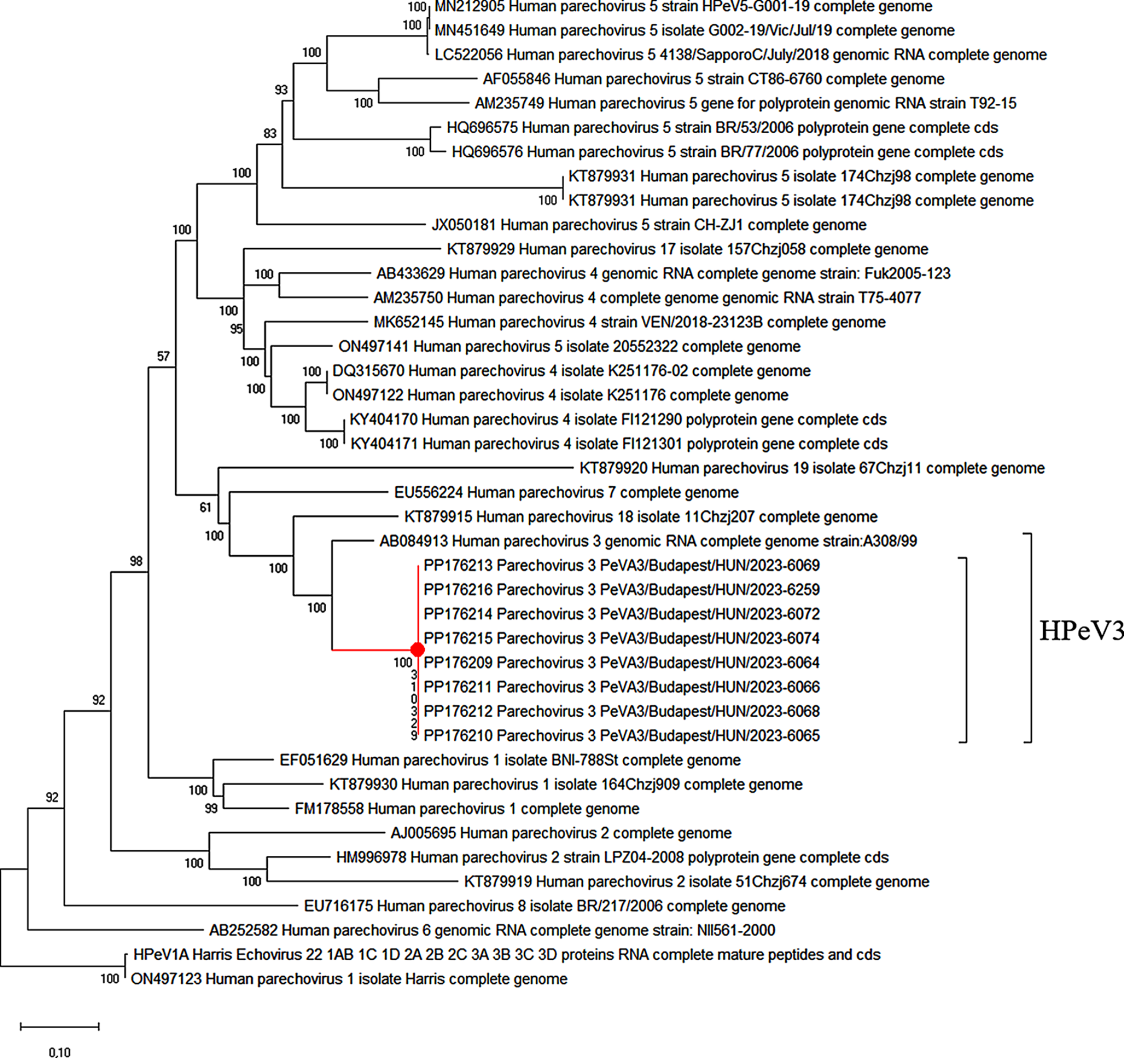
Phylogenetic tree based on the complete coding region using the GTR + G + I model. The analysis included the eight whole genome sequences of the present study and 33 sequences and reference sequences representing global PEV-A strains derived from the Genbank database. Study sequences clustered together and were allocated to PEV-A genotype 3. Scale bars indicate nt substitutions per site and bootstrap values on the branches were calculated using 1000 replicates

According to the phylogenetic classification, all eight PEV-A3 complete genome sequences (PP176209 – PP176216) obtained in this study clustered together and were allocated to PEV-A genotype 3 supported by a 100% bootstrap value (Fig. 3). The closest relation was shown with the PEV-A3 reference sequence published in 2004 (Genbank accession number AB084913) [30].

Phylogenetic analysis of the VP1 (Supplementary Figure S1a) showed that the consensus sequence of the present study clustered together with a strain detected in Poland in March 2019 (Genbank accession number OM895625) sharing 99.2% pairwise identity at the nt level with an average *p*-distance of 0.01. We also investigated the genetic relationship between the study sequences and previously described Hungarian PEV-A3 sequences based on the VP1 (Genbank accession number MH845214) and VP3 (Genbank accession numbers MH845197, MH845198, MH845199, MH845200, MH845201, MH845202, MH845203 and MH845204) regions (Supplementary Figure S1b). According to the phylogenetic analysis and the mean pairwise identity in the VP1 (96.8%) and VP3 (97.6%) regions, complemented with an average *p*-distance of 0.03 (SE ± 0.01), our results suggest a relatively distinct relationship between the current and previously described Hungarian PEV-A3 strains.

To investigate potential recombination events and the genetic relationship between the P1, P2 and P3 coding regions, we performed phylogenetic analysis based on the consensus study sequence and highly similar PEV-A3 sequences (*n* = 91) available in Genbank (Supplementary Figure S2). According to the generated phylogenetic trees, the consensus study sequences clustered with PEV-A3 strains isolated in Yamagata, Japan in 2019 (Genbank accession numbers LC652472, LC652473, LC652474, LC652475, LC652476, LC652478, LC652480 and LC652481) and were monophyletic with an Australian recombinant (AR) strain isolated in Queensland in 2020 (Genbank accession number MW076475), for all three regions (Supplementary Figure S2a-c) [31]. Overall mean identity between the consensus sequences and the Yamagata strains was 98.7%, 98.5% and 98.4% at the nucleotide level in the P1, P2 and P3 regions, respectively, and 99.1%, 99.5% and 99.1% at the amino acid level. Furthermore, the study consensus sequence showed similarity (98.7% at the nucleotide level) with partial US sequences limited only to the P1 capsid region, isolated in 2019 (Genbank accession numbers MN896922 and MN896931).

The average *p*-distance between the study sequence and other PEV-A3 strains was similar in the P2 and P3 non-structural regions compared to P1 structural region (0.02 SE ± 0.0 for all regions), indicating homogeneous divergence and relative conservation in all regions. Furthermore, we found no topological inconsistencies between the P1 capsid and P2, P3 non-capsid regions based on the phylogenetic analysis. The similarity plotting performed on the consensus study sequence against closely related PEV-A strains selected based on Fig. 1 and Supplementary Figure S2 also confirmed the lack of recombination events after the emergence of the AR strains (Supplementary Figure S3).

## Discussion

The present study reports the detection of PEV-A3 in Hungary with the first complete genome sequences and genome analysis. The confirmed positive cases are linked to a maternity unit of a single hospital in Hungary. Although it is known that PEV-A is a circulating pathogen in the country [19, 20], this is the first time that whole genome sequences of persisting PEV-A3 strains have been directly obtained from clinical samples in association with severely ill patients.

In the present outbreak, affected neonates presented high fever, sepsis-like symptoms and three of them showed CNS involvement. PEV-A3 infection has the most severe clinical manifestations in comparison to other genotypes, which is assumed to be linked to the increased neural tropism and low levels of neutralizing antibodies produced against this virus genotype [11].

Microbiological diagnostic procedures of PEV-A infection include nucleic acid detection from stool and/or respiratory samples of the patient by RT-qPCR targeting the 5’ UTR, which is well-conserved among all PEV-As [9, 10]. When comparing Ct values of positive stool and swab samples from the same patient, we found that stool samples showed lower Ct values, indicating higher virus concentration. It is also important to note, however, that the virus was detected in the fecal sample of one asymptomatic mother, although her pharyngeal swab specimen was negative. According to the literature, the virus can be shed in the feces for up to several months, and therefore, most epidemiological studies on PEV-A are performed on stool samples from infants with acute symptoms [4, 7, 11]. In cases of CNS involvement, we found that CSF samples are suitable for PEV-A diagnosis as well. Virus isolation was also attempted unsuccessfully in Vero, Vero E6, RD and A549 cells from PEV-A-positive samples; however, it is hypothesized that propagation of PEV-A3 is less sensitive especially in RD cells compared to other PEV-A genotypes [11].

As only partial viral genome sequences were reconstructed with metagenomic sequencing, and to confirm that the cases are microbiologically related, a more comprehensive genome analysis was performed on a total of nine samples of all five symptomatic neonates using amplicon-based whole genome sequencing. At the consensus level, whole coding sequences were identical suggesting that the cases were microbiologically linked. We found two ambiguities in the VP1 region in gene positions 119 (G > U, resulting Gly to Val change in VP1 aa position 40) and 123 (U > A, synonymous mutation) at frequencies of 17.7–48.0% and 22.0–53.7%, respectively, indicating the simultaneous presence of at least two quasispecies. These two nucleotide variations were found in all sequences, but at a higher frequency in the sample of Neonate 5, inferring that Neonate 5 was probably the first case, followed by the infection of the other four neonates.

The phylogenetic classification of the complete coding region showed that the generated sequences were allocated to PEV-A3 and clustered with the reference strain AB084913. The phylogenetic analysis based on the VP1 showed that the consensus sequence of the present study clustered together with a Polish strain detected in March 2022, suggesting that the PEV-A3 strain causing the current outbreak had been circulating in the Eastern European region for the last years. According to a systematic survey evaluating parechovirus circulation and testing capacity in Europe between 2015 and 2021, the most frequently reported genotype in this region was PEV-A3 [16]. We also investigated the genetic relationship between the study sequences and the previously described Hungarian PEV-A3 sequences based on the VP1 and VP3 regions [19, 20]. Due to the relatively distinct relationship between the strains, it is assumed that the current epidemic was not related to the previously detected and described Hungarian PEV-A3 strains [19, 20]. Phylogenetic analysis of the P1, P2 and P3 regions showed that the consensus study sequences clustered together with strains isolated in Yamagata, Japan in 2019 and were monophyletic with Australian recombinant (AR) strains isolated in 2020 [31, 32]. Mizuta *el al.* analyzed the sequences of multiple PEV-A3 cases in Japan that formed two clusters; cluster 2019 A was replaced by 2019B in the autumn of 2019. A phylogenetic analysis based on the non-structural protein regions revealed that 2019B was closely related to the AR strain, suggesting that the ancestor to the P2 and P3 regions of this cluster were recombinant [31]. The AR strain emerged in 2013 and coincided with a biennial outbreak of sepsis-like infections in infants, showing a decreasing prevalence in the 2019–2020 season, but this strain was not associated with increased severity compared to the clinical outcome of infections caused by non-recombinant PEV-A3 strains [32]. We found that the average *p*-distance of the study sequences from other PEV-A3 strains was similar in the P1 capsid, and P2 and P3 non-structural regions (mean *p*-distance 0.02 SE ± 0.00) suggesting homogenous diversity in all three regions. We also found no topological inconsistencies between the P1, P2 and P3 based on the phylogenetic and similarity plotting analyses either, inferring that recombination events between the presented PEV-A3 strains and other PEV-A strains during evolution since the previously described progressive evolution of AR strains [32] were not likely. This is supported by published data showing that PEV-A3 is less prone to recombine, and is phylogenetically distinct from other PEV-A types [33]. Although PEV-A3 is equally divergent in nucleotide sequence as other genotypes, the genetic distinction may reflect different cellular tropism arising from the missing arginine–glycine–aspartic acid (RGD) motif in the VP1 C terminus and use of a non-integrin receptor during infection [11, 33].

In the present outbreak, the outbreak investigation team retrospectively considered that droplet/airborn transmission was the most likely, while contact transmission could not be excluded either. From the epidemiological investigation, it is worth highlighting that albeit the mother of Neonate 5 – the first neonatal case presumed by the results of whole genome sequencing – was asymptomatic during her stay in Hospital X, family members living in the same household had respiratory symptoms in her peripartum period and also visited her post-delivery. When taking the medical history of women admitted for delivery, it is recommended to record whether there are any symptomatic household members, and, if so, care and patient placement options should be considered accordingly. Hospital policies for visitors should also reflect the risks of pathogen transmission, especially during the respiratory virus season.

To summarize, this is the first time that complete genome sequencing and phylogenetic characterization of circulating PEV-A3 strains have been performed in Hungary, suggesting that the outbreak cases were microbiologically linked. As proposed by Bubba et al., results on parechovirus infections and epidemiology suggest an urgent need to introduce and expand diagnostics and genotyping capacity [16]. Furthermore, integrating parechoviruses with non-polio enterovirus surveillance would help characterize and better undestand the circulation and seasonality of parechoviruses across Europe and worldwide [16]. Our findings can facilitate future investigations on the evolution and pathogenicity of PEV-A3 infections as well as differential diagnosis from other enterovirus-like infections, and provide resources for identifying and typing parechovirus infections.

## Electronic supplementary material

Below is the link to the electronic supplementary material.

Supplementary Material 1

## Data Availability

The Genbank accession numbers for all newly generated sequences assembled in this outbreak investigation study are: PP176209 – PP176216 (complete consensus sequences) and PP176217 (partial genome sequence).
