# Supplementary material for: First whole genome sequencing and analysis of human parechovirus type 3 causing a healthcare-associated outbreak among neonates in Hungary
Source: Eur J Clin Microbiol Infect Dis. 2024 Sep 27;43(12):2341–50. doi: 10.1007/s10096-024-04950-4 (PMC11608160; doi:10.1007/s10096-024-04950-4)
Supplement: Supplementary file 1 — Supplementary Material 1 [file 10096_2024_4950_MOESM1_ESM.docx]

**Supplementary materials**

**Title: First whole genome sequencing and analysis of human Parechovirus type 3 causing a healthcare-associated outbreak among neonates in Hungary**

Nóra Deézsi-Magyar^a,e^, Nikolett Novák^a^, Adrienne Lukács^a^, Katalin Réka Tarcsai^a^, Ágnes Hajdu^b^, László Takács^c^, Ferenc Balázs Farkas^d,e,f^, Zita Rigó^a^, Erzsébet Barcsay^a^, Zoltán Kis^f,a^* and Katalin Szomor^a^*

^a^ Department of Microbiological Reference Laboratories, National Center for Public Health and Pharmacy, Budapest, Hungary

^b^ Department of Communicable Disease Epidemiology and Infection Control, National Center for Public Health and Pharmacy, Budapest, Hungary

^c^ Bethesda Children Hospital, Budapest, Hungary

^d^ Pediatric Center, Semmelweis University, Budapest, Hungary

^e^ School of PhD Studies, Semmelweis University, Budapest, Hungary

^f^ Institute of Medical Microbiology, Faculty of Medicine, Semmelweis University, Budapest, Hungary

*Corresponding authors: Dr. Zoltán Kis, e-mail: [kis.zoltan@semmelweis.hu](mailto:kis.zoltan@semmelweis.hu)

Address: Albert Flórián Rd. 2-6. 1097, Budapest, Hungary

**Supplementary Table S1.** List and binding sites of the custom-designed sequencing primers applied during this investigation; primers were used in two pools in equimolar concentration for target amplification (National Reference Laboratory for Enteroviruses, National Center for Public Health and Pharmacy, Budapest, Hungary).

| Primer | Pool | Sequence (5' - 3') | Size | %GC | Position (bp) |
| --- | --- | --- | --- | --- | --- |
| hPeV-3_1_LEFT | 1 | ATACCCCGATTTGCTGAGCTTC | 22 | 50.00 | 41 – 987 |
| hPeV-3_1_RIGHT | 1 | GCCATTGAATGAAAGTTATCCACATTTC | 28 | 35.71 |  |
| hPeV-3_2_LEFT | 2 | GATGTAGTGCAAGCTACGACCA | 22 | 50.00 | 899 – 1895 |
| hPeV-3_2_RIGHT | 2 | CTACTGTGGCTGCCCAATCAAA | 22 | 50.00 |  |
| hPeV-3_3_LEFT | 1 | TGATCCTAGAACTGCAGGGAGT | 22 | 50.00 | 1750 – 2745 |
| hPeV-3_3_RIGHT | 1 | TCTTCAGTGTCATATGTATGAGCCAC | 26 | 42.31 |  |
| hPeV-3_4_LEFT | 2 | AAGAGGGTCATGGCATGTTGTC | 22 | 50.00 | 2625 – 3631 |
| hPeV-3_4_RIGHT | 2 | TGTAGACAAACAAGCAGTGGTTAGA | 25 | 40.00 |  |
| hPeV-3_5_LEFT | 1 | TCCTCAGCAGCCACAGAAATTC | 22 | 50.00 | 3500 – 4487 |
| hPeV-3_5_RIGHT | 1 | TCTCTTCAAGATGTGCCATTGGG | 23 | 47.83 |  |
| hPeV-3_6_LEFT | 2 | GCCAGTGAGTTCATGGATGGTT | 22 | 50.00 | 4340 – 5339 |
| hPeV-3_6_RIGHT | 2 | GCTGGTATCCTGCACAATGTGT | 22 | 50.00 |  |
| hPeV-3_7_LEFT | 1 | GCCAAACCAAAGAGTGCTTTCC | 22 | 50.00 | 5192 – 6188 |
| hPeV-3_7_RIGHT | 1 | CGGACTTAACAAAGCTGTACCCT | 23 | 47.83 |  |
| hPeV-3_8_LEFT | 2 | GGCAAGGTGTTAAAGCATGTGTC | 23 | 47.83 | 6039 – 7057 |
| hPeV-3_8_RIGHT | 2 | GCTGCTTGAATGTGCTGAAGTTT | 23 | 43.48 |  |
| hPeV-3_9_LEFT | 1 | ACCACCATCTTTAACACTTGTCTCA | 25 | 40.00 | 6296 – 7323 |
| hPeV-3_9_RIGHT | 1 | TGGTATGTCCAATATTCCAAATTAGTGTTC | 30 | 33.33 |  |

**Supplementary Table S2.** Samples and assembly metrics data of the amplicon-based whole genome sequencing. *Full-length genome size was determined between the binding site of the hPeV-3_1_LEFT primer (nt position 41) and the 3’-poly(A) tail. **Only partial PEV-A3 genome sequence was obtained from this sample.

| **Patient** | **Sample type (sample ID)** | **Ct value** | **Sequence length (bp)*** | **Average sequencing depth** | **Number of allocated reads** | **Genbank accession number** |
| --- | --- | --- | --- | --- | --- | --- |
| Newborn 1 | stool (6074) | 31.11 | 7 293 | 12 589 | 646 845 | PP176215 |
|  | Nasal swab (6072) | 31.55 | 7 293 | 13 219 | 693 302 | PP176214 |
| Newborn 2 | Stool (6068) | 29.64 | 7 293 | 14 432 | 691 019 | PP176212 |
|  | Nasopharyngeal swab (6069) | 32.71 | 7 292 | 13 751 | 766 396 | PP176213 |
| Newborn 3 | Stool (6064) | 30.65 | 7 293 | 20 492 | 990 133 | PP176209 |
|  | Nasopharyngeal swab (6065) | 30.90 | 7 293 | 11 784 | 604 499 | PP176210 |
| Newborn 4 | Stool (6066) | 33.07 | 7 293 | 8 324 | 443 404 | PP176211 |
|  | CSF (6148)** | 32.1 | 6424 | 6 952 | 410 200 | PP176217 |
| Newborn 5 | Nasopharyngeal swab (6259) | 32.11 | 7 293 | 9 840 | 557 397 | PP176216 |

**Supplementary Figure S1.** Phylogenetic tree constructed based on the **a**. VP1 and **b**. VP3 regions using the T92+G model including the consensus sequence of PP176209 – PP176217 (red dots) and other sequences representing genetically close strains derived from the Genbank database. Previously described Hungarian sequences are highlighted with black dots. Scale bars indicate nt substitutions per site. Bootstrap support values were calculated using 1000 replicates. PEV-A1 reference sequence ECHPICORN Echovirus 22 (Genbank accession number L02971) was set as outgroup.


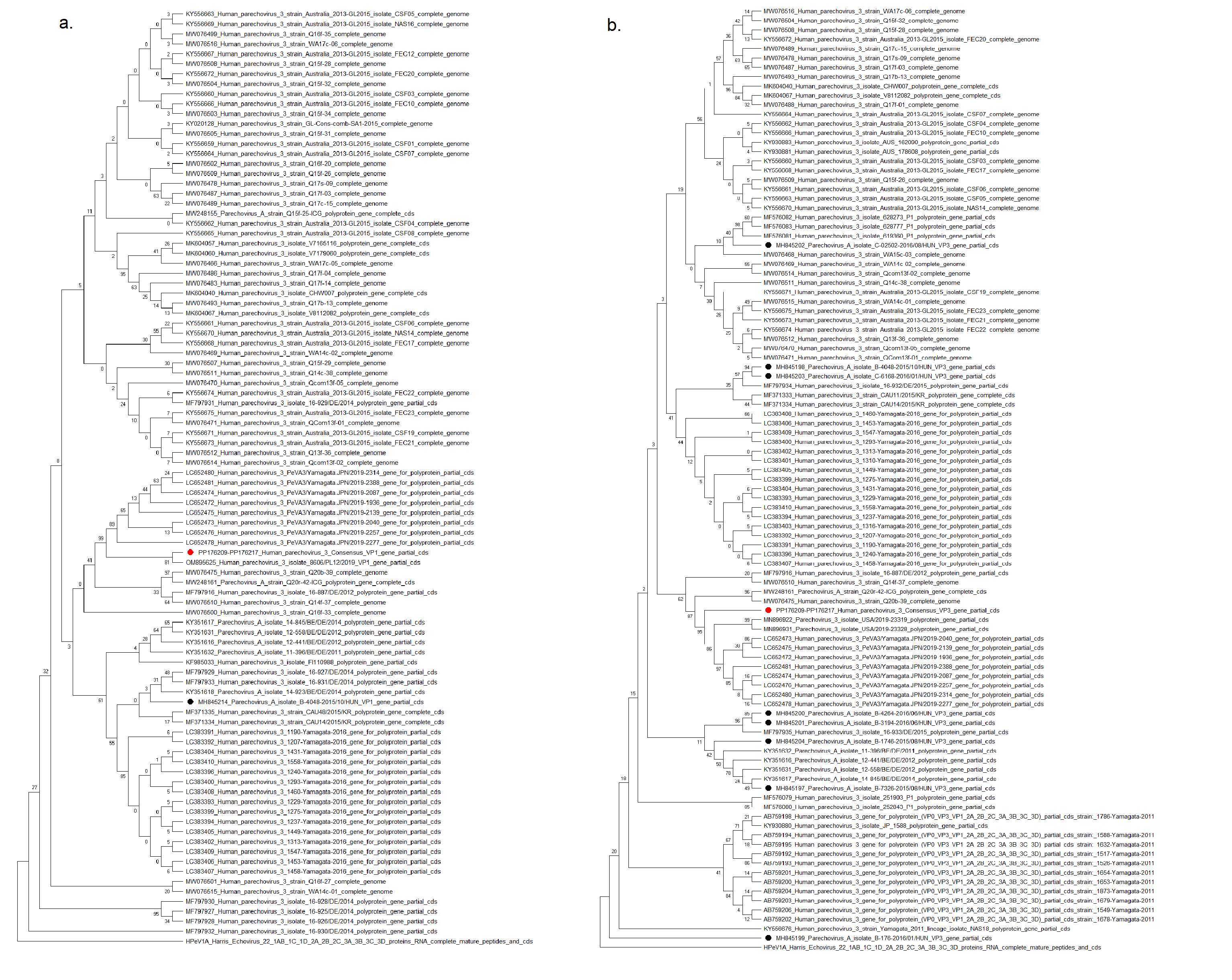


**Supplementary Figure S2.** Phylogenetic tree of the P1 capsid, and the P2, P3 non-structural regions (GTR+G+I model) including the consensus sequence of PP176209 – PP176216 and 91 sequences representing genetically close strains derived from the Genbank database. **a.** P1 capsid region, **b.** P2 non-structural region, **c.** P3 non-structural region. Scale bars indicate nucleotide substitutions per site. Bootstrap support values were calculated using 1000 replicates. PEV-A1A reference sequence ECHPICORN Echovirus 22 (Genbank accession number L02971) was set as outgroup.


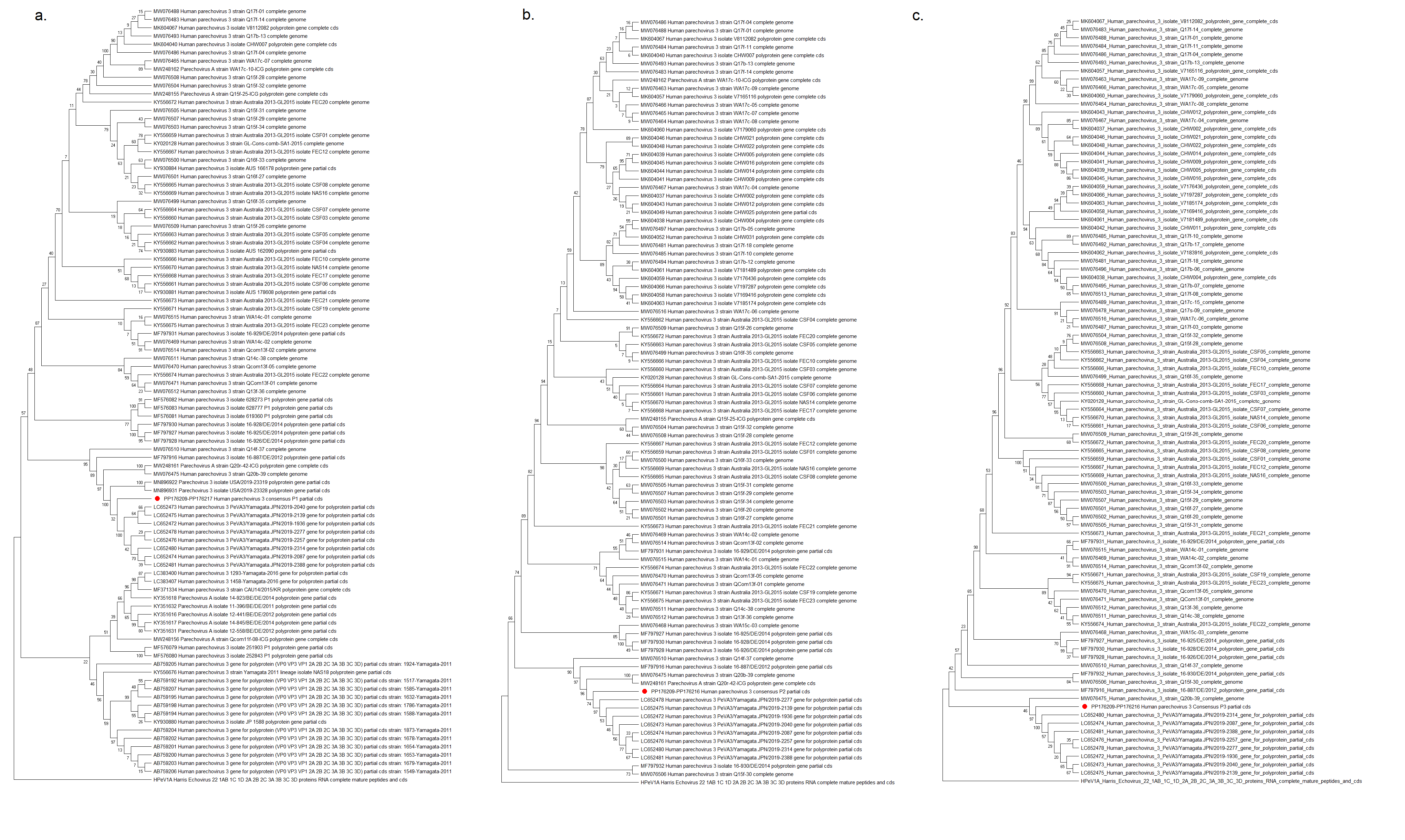


**Supplementary Figure S3.** Plot of similarity of the study PEV-A3 strains (represented with strain PP176214) and closely related strains. Sequence AB084913 (PEV-A3) was set as reference. Analysis was conducted by using SimPlot++ v1.3 (Kimura distance model, window size 200 bp moving in 20 nt steps).

**
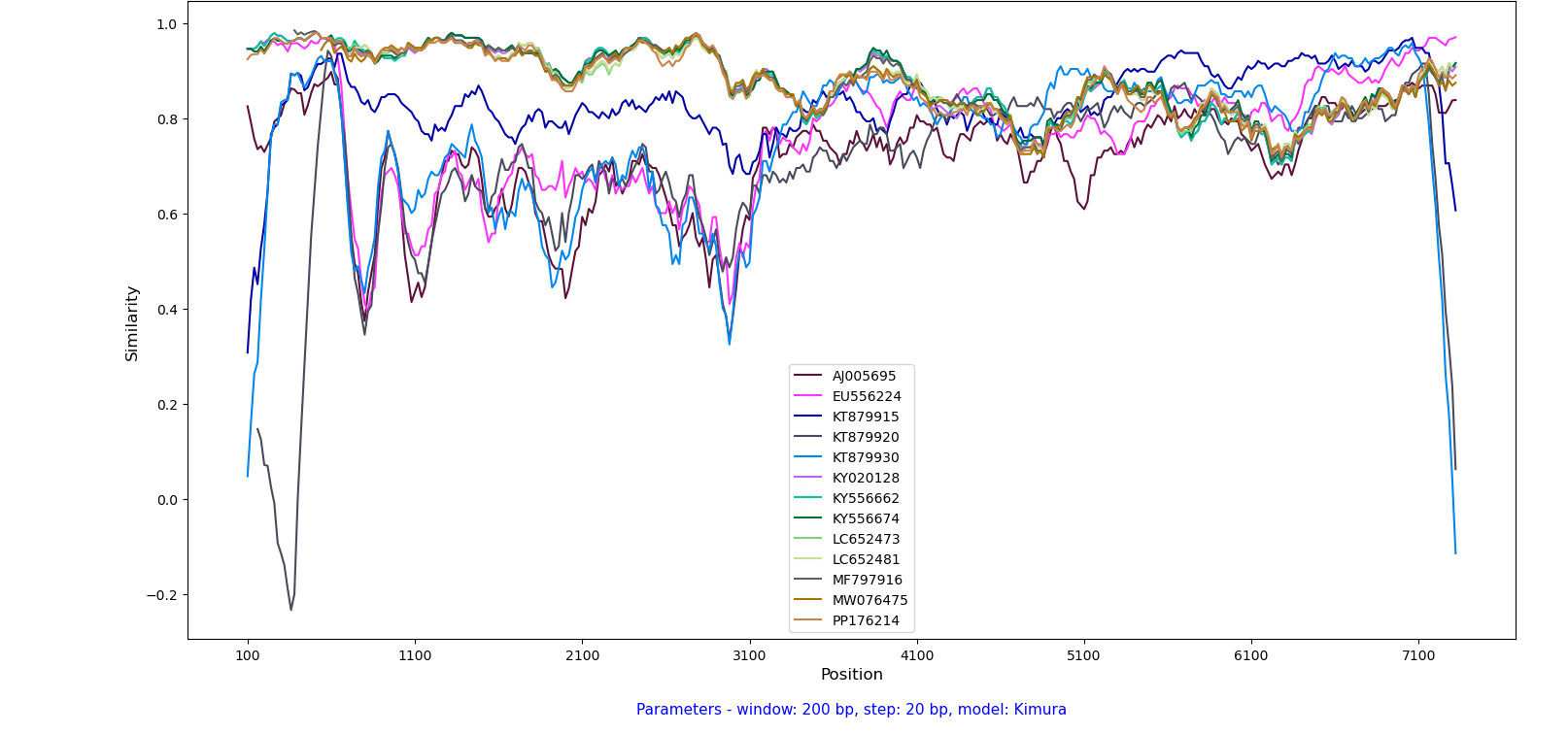
**
